# Effects of high light exposure and heterologous expression of β-carotene ketolase on the metabolism of carotenoids in *Chlamydomonas reinhardtii*

**DOI:** 10.3389/fbioe.2025.1533661

**Published:** 2025-03-10

**Authors:** Rui Mei, Haihong Yang, Chunli Guo, Zeyu Hong, Zhangli Hu, Yan Wu, Danqiong Huang, Chaogang Wang

**Affiliations:** ^1^ Shenzhen Key Laboratory of Marine Bioresource and Eco-Environmental Science, College of Life Sciences and Oceanography, Shenzhen University, Shenzhen, China; ^2^ Guangdong Technology Research Center for Marine Algal Bioengineering, College of Life Sciences and Oceanography, Shenzhen University, Shenzhen, China; ^3^ Instrumental Analysis Center, Shenzhen University, Shenzhen, China

**Keywords:** carotenoids, canthaxanthin, *Chlamydomonas reinhardtii*, heterologous expression, metabolic analysis

## Abstract

**Introduction:**

Stress from high light exposure and overexpression of β-carotene ketolase can have significant effects on the synthesis of carotenoids in *Chlamydomonas reinhardtii*. As a promising platform for carotenoid production, *C. reinhardtii* needs further research and technological innovation to address challenges, such as environmental interference, exogenous gene expression, and metabolic regulation, to achieve efficient and sustainable production of carotenoids.

**Methods:**

Appropriate β-carotene ketolase were selected from different organisms and subjected for codon optimization based on the preferences of the nuclear genome of *C. reinhardtii*. After designation, including intron insertion and chloroplast transit peptide, expression vectors were constructed and used for nuclear transformation of *C. reinhardtii* CC849 by bead milling method. Subsequently, DNA-PCR and RT-PCR were used to identify positive transformants grown with antibiotic stress, LC-MS/MS and metabolic analysis were performed to evaluate the products of transformants.

**Results:**

In this study, carotenoid metabolism regulation in C. reinhardtii was investigated in a time-dependent manner through high light exposure and heterologous expression of β-carotene ketolase. The results suggested that the stress from high light exposure (500 μmol/m^2^/s) negatively regulated the accumulation of β-carotene; positively induced the accumulation of zeaxanthin, echinenone, and canthaxanthin; and continuously promoted accumulation of zeaxanthin and canthaxanthin in *C. reinhardtii*. Metabolomics analysis suggested that high light exposure stress promoted biosynthesis of carotenoids, improved the intermediates associated with the astaxanthin synthesis pathway, and promoted conversion of β-carotene to downstream substances. Several strategies were implemented to improve canthaxanthin production in *C. reinhardtii* to achieve overexpression of β-carotene ketolase genes from different sources, including strong promoters, insertion introns, and chloroplast conduction peptides. It was found that β-carotene, echinenone, and canthaxanthin were all significantly increased in the transformed *C. reinhardtii* overexpressing β-carotene ketolase. Among these, the highest canthaxanthin content was found in pH124-CrtO, which was seven times that observed in the wild type. Moreover, the metabolomics analysis of carotenoids showed promotion of the abscisic acid and astaxanthin pathways in the transformed *C. reinhardtii*.

**Discussion:**

The results of this study provide a new scheme for manipulating the metabolism of carotenoids and promoting the synthesis of high-value carotenoids in *C. reinhardtii*.

## 1 Introduction

**FIGURE 1:**
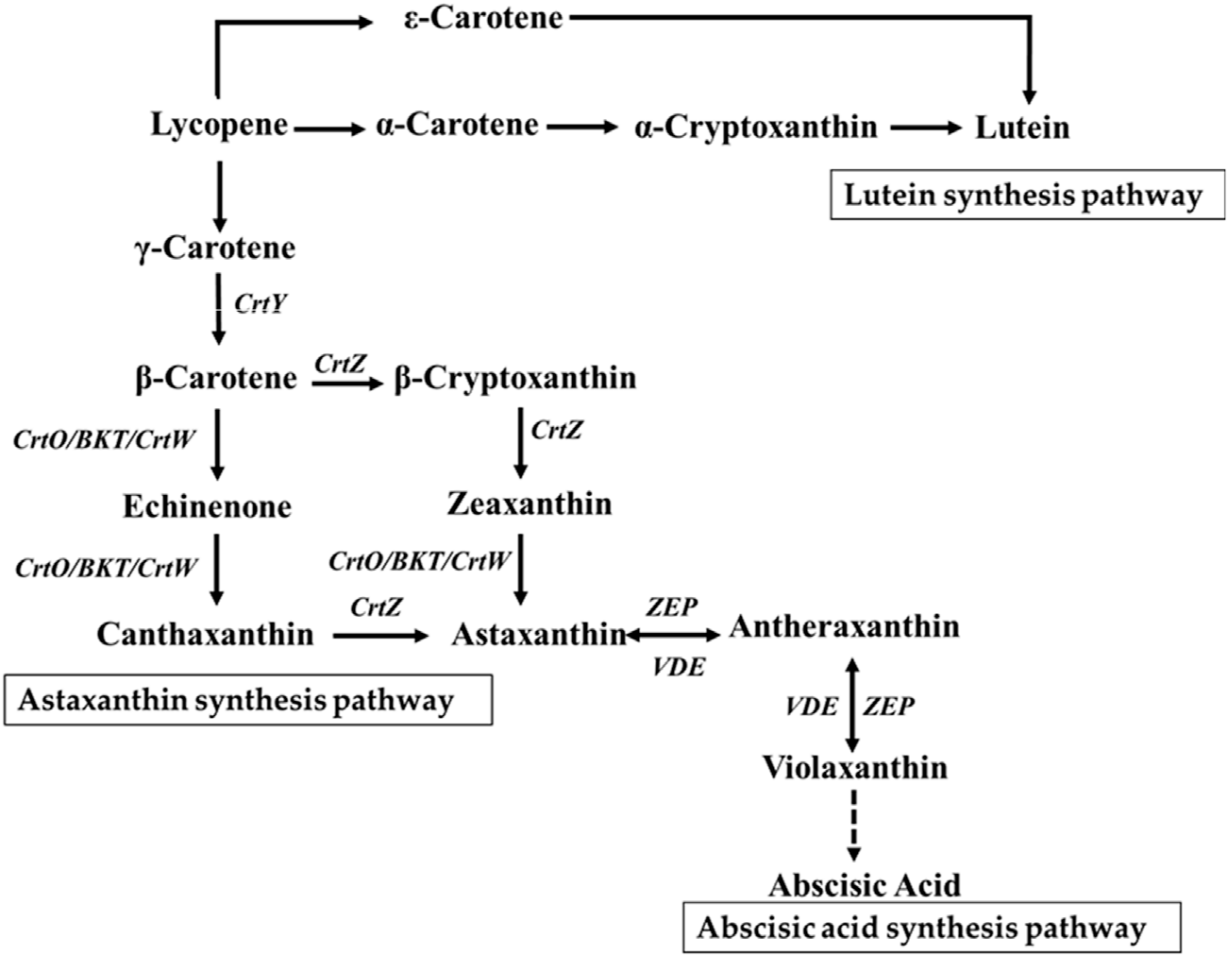
Carotenoid metabolic pathway. *CrtY*, lycopene cyclase; *CrtW*, β-carotene ketolase from bacteria; *CrtO/BKT*, β-carotene ketolase from algae; *CrtZ*, β-carotene hydroxylase from bacteria; *ZEP*, zeaxanthin epoxidase; *VDE*, violaxanthin de-epoxidase.

Carotenoids are some of the widely distributed terpenoid derivatives in nature. Based on their chemical structures, carotenoids are divided into two major categories as carotenes and xanthophylls (Maoka, 2009). The carotene types include α-carotene, β-carotene, and lycopene, while the xanthophyll types can be further categorized into two branches as α-branch and β-branch. The α-branch comprises derivatives of α-carotene, such as lutein, whereas the β-branch mainly comprises derivatives of β-carotene, such as zeaxanthin, antheraxanthin, echinenone, canthaxanthin, violaxanthin, and astaxanthin (Liu et al., 2024) (Figure 1). Carotenoids function as accessory pigments in chloroplast photosynthesis and protect the chloroplasts from damage caused by intense light exposure (Young, 1991). Studies have shown that exposure to high-intensity light increases the carotenoid content in microalgae, such as *Haematococcus pluvialis* (Liyanaarachchi et al., 2020), *Dunaliella salina* (Wu et al., 2020), *Chlorella zofingiensis* (Sun et al., 2019), and *Botryococcus braunii* (Khajepour et al., 2015), with variations in carotenoid compositions among the different microalgal species (Solovchenko, 2015). When Cyanobacteria were grown under high light exposure, specific carotenoids were found to be accumulated in large quantities, particularly zeaxanthin and echinenone (Steiger et al., 1999). In *Chlamydomonas*, both carotenoid and phenolic compound levels were found to be elevated following exposure to high light intensity (Faraloni et al., 2021). In addition to their role in light harvesting, carotenoids possess excellent antioxidant activities. Therefore, high-value carotenoids such as astaxanthin and canthaxanthin that have distinct coloration and health benefits are widely used in human nutraceutical, cosmetic, and animal feed industries (Bjørklund et al., 2022).

Canthaxanthin is a type of ketocarotenoid derived from β-carotene through two ketolation reactions on the β-ionone rings (Alvarez et al., 2006); it is primarily found in microalgae (Han et al., 2013), fungi (Visser et al., 2003), bacteria (Ide et al., 2012), certain flowering plants (Cunningham and Gantt, 2011), and protozoa (Watanabe et al., 2018). Studies indicate that canthaxanthin is one of the most potent natural antioxidant pigments whose capacity far exceeds that of β-carotene or vitamin E (Venugopalan et al., 2013); it effectively scavenges reactive oxygen species (ROS) and eliminates free radicals. Canthaxanthin has been widely used as a colorant, nutrient, and antioxidant in products such as food, pharmaceuticals, nutraceuticals, animal feed, and cosmetics (Ray et al., 2012; Chitchumroonchokchai and Failla, 2017). Given the high commercial value of the nutraceutical market and its growing demand, several methods have been developed for the large-scale production of natural canthaxanthin in a more sustainable manner as an alternative to synthetic production. Additionally, regulation of the carotenoid biosynthesis pathway through genetic engineering allows production of high-valued carotenoids like canthaxanthin in heterologous systems (Nunes et al., 2021). The biosynthesis of canthaxanthin has been successfully achieved in yeast (Tramontin et al., 2019), bacteria (Scaife et al., 2012), plants (Huang et al., 2013), and microalgae (Tran and Kaldenhoff, 2020). It is well known that β-carotene ketolase is the key enzyme in the synthesis of canthaxanthin, and it catalyzes the conversion of β-carotene into canthaxanthin. In organisms that accumulate canthaxanthin, β-carotene ketolase is encoded by different genes. For example, it is encoded by the *CrtW* gene in bacteria (Scaife et al., 2009) and *CrtO* (or *BKT*) gene in algae (Zhang et al., 2020). The cytochrome P450 enzyme encoded by the *CrtS* gene in the fungus *Xanthophyllomyces dendrorhous* functions as both β-carotene ketolase and hydroxylase (Alvarez et al., 2006). Through genetic modifications of the β-carotene ketolase gene, canthaxanthin synthesis has been successfully achieved in fungi (Papp et al., 2013), bacteria (Scaife et al., 2012), and plants (Zhu et al., 2018).

*Chlamydomonas reinhardtii* is a eukaryotic organism with a fully enclosed nuclear membrane; the integration of exogenous genes into its nuclear genome follows either the homologous recombination or non-homologous end joining (NHEJ) pathways (Kindle et al., 1989). The efficiency of exogenous gene integration into the nuclear genome of *C. reinhardtii* can be enhanced using strong promoters, codon optimization, intron sequences, and appropriate terminator selection (Zhang et al., 2021). Currently, stable genetic transformation systems have been established in the nucleus, chloroplast, and mitochondrion of *C. reinhardtii*. To date, many functional enzymes (Yoon et al., 2011), antibodies (El-Ayouty et al., 2019), and vaccines (Rasala and Mayfield, 2015) have been successfully expressed in *Chlamydomonas*. The chloroplast of *C. reinhardtii* is rich in β-carotene and provides an efficient cellular factory for the synthesis of canthaxanthin (Li et al., 2012). Compared to yeast and plants, the culture conditions of *C. reinhardtii* are simple and inexpensive. The reproduction rate of *C. reinhardtii* is high, and the associated large-scale culture technology is mature. *C. reinhardtii* has been approved in the novel food catalog and can be consumed directly (Chen et al., 2023). Therefore, the insertion and expression of β-carotene ketolase in the chloroplast of *C. reinhardtii* through genetic engineering approaches has become popular for the synthesis of high-value carotenoids like canthaxanthin.

The present study is concerned with regulation of carotenoid synthesis in *C. reinhardtii* through high-intensity light stimulation and heterologous expression of β-carotene ketolase. First, metabolomics analysis was employed to examine the dynamic changes in carotenoid composition in *C. reinhardtii* under high-intensity light stress. Then, several strategies were compared to obtain engineered strains with significantly increased canthaxanthin content and efficiently express β-carotene ketolases driven by different organisms in *C. reinhardtii*; these schemes include promoter selection, introduction of *RbcS2* intron1, and chloroplast transit peptide. The results of this study provide a foundational basis for utilizing *C. reinhardtii* as a cellular factory to synthesize high-value carotenoids with enhanced nutritional efficacies.

## 2 Materials and methods

### 2.1 Culture of bacterial and algal strains

*Escherichia coli* DH5α (Yeasen Biotechnology Co., Ltd., Shenzhen, China) was used to construct and propagate recombinant plasmids. The *E. coli* cells were cultured at 37°C and 250 rpm in liquid or solid LB medium (5 g/L of yeast extract, 10 g/L of peptones, 10 g/L of NaCl, and additional 15 g/L of agar for the solid medium) containing 100 μg/mL of ampicillin (Biosharp, Anhui, China). CC849 is a cell-wall-deficient strain of *C. reinhardtii* (purchased from the *C. reinhardtii* Resource Center and maintained at the Guangdong Technology Research Center for Marine Algal Bioengineering, Shenzhen University) that was cultured in liquid or solid TRIS acetate phosphate (TAP) medium (https://www.chlamycollection.org/methods/media-recipes/tap-and-tris-minimal/) containing 100 μg/mL of ampicillin (Biosharp, Anhui, China) at 25°C with 16 h of light exposure at 100 μmol/m^2^/s and 8 h in the dark. Transgenic algal strains were selected on a medium supplemented with appropriate antibiotics [100 μg/mL of ampicillin + 10 μg/mL of zeocin (Intensity, CA, United States)].

### 2.2 High-intensity light treatment

High-intensity light treatments of *C. reinhardtii* CC849 and the transformants were performed as reported previously (Perozeni et al., 2020). Briefly, a single colony of *C. reinhardtii* CC849 was inoculated in a 50-mL Erlenmeyer flask containing 20 mL of TAP medium; this culture was grown in an incubator at 25°C under 16 h of light exposure at 100 μmol/m^2^/s and 8 h in the dark for 2–3 d until the cell density reached approximately 2 × 10^6^ cells/mL. The cells were then diluted to a concentration of 5 × 10^5^ cells/mL and inoculated in 100-mL Erlenmeyer flasks containing 50 mL of fresh TAP medium with at least three replicates. The inoculated cultures were continuously exposed to light at 25°C and 100 μmol/m^2^/s for 2 days, followed by exposure to high-intensity light at 500 μmol/m^2^/s for the next 7 days. Samples were collected every day, and the algal cells were harvested by centrifugation at 5,000 rpm and room temperature for 10 min. The algal cells were then dried in a vacuum freeze dryer for 48 h and weighed; this dry mass was expressed in terms of grams per liter.

### 2.3 Plasmid design and construction

**FIGURE 2:**
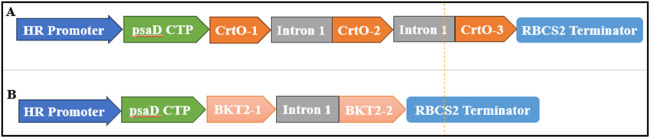
Diagram of the expression vectors of **(A)** pH124-CrtO and **(B)** pH124-BKT2. HR promoter, *HSP70A/RBCS2* promoter; psaD CTP, psaD chloroplast transit peptide; Intron 1, *RBCS2* intron 1; *CrtO*-1*, CrtO*-2, and *CrtO*-3, β-carotene ketolases from *C. reinhardtii*; *BKT2*-1*, BKT2*-2, and *BKT2*-3, β-carotene ketolases from *H. pluvialis*; RBCS2 Terminator, 3′UTR of *RbcS2* gene in *C*. *reinhardtii*.

The β-carotene ketolase used in this study included *CrtO* from *C. reinhardtii* (GenBank: AY860820.1) and *BKT2* from *H. pluvialis* (GenBank: D45881.1). These genes were codon-optimized based on the codon preference of *C. reinhardtii*. Additionally, *RbcS2* intron1 (GenBank: X04472.1) was inserted at the coding regions of *CrtO* and *BKT2*. A chloroplast transit peptide (cTP) (GenBank: Q39615.1) was then inserted at the N-terminal ends of *CrtO* and *BKT2*. The pH124 vector carrying the zeocin resistance gene was used as the backbone, which resulted in the pH124-CrtO and pH124-BKT2 plasmids (Figures 2A, B).

### 2.4 Genetic transformation of *C. reinhardtii* and transformants identification

The nuclear transformation of *C. reinhardtii* CC849 was carried out using the glass bead transformation method (Li et al., 2021). Here, colonies grown on TAP solid plates containing 100 μg/mL of ampicillin and 10 μg/mL of zeocin were subjected to the genomic DNA polymerase chain reaction (PCR) and reverse transcription polymerase chain reaction (RT-PCR) analyses for molecular identification. First, the transformed cells were transferred to new TAP solid plates containing 100 μg/mL of ampicillin and 10 μg/mL of zeocin for culturing over 1 week. Genomic DNA extraction from the algal cells was performed using the Ultra DNA Isolation Kit from Bebebio (Beibei Biotechnology Co., Ltd., Zhengzhou, China). For the pH124-CrtO and pH124-BKT2 transgenic algal transformants, colony PCR was performed with the primer sets CrtO-F/R and BKT2-F/R, respectively (Table 1). PCR amplification was then carried out using the 2× M5 HiPer plus Taq HiFi PCR mix (with blue dye) (Mei5 Biotechnology Co., Ltd., Beijing, China) under the following program: 95°C for 3 min; 35 cycles at 94°C for 25 s, 60°C for 25 s, and 72°C for 1 min, with a final extension at 72°C for 5 min. For the RT-PCR, the total RNA was extracted using the RNA fast 200 Kit (Fastagen, Shanghai, China), and 1 μg of the total RNA was reverse transcribed to cDNA using the Hifair^®^ III 1st Strand cDNA Synthesis SuperMix for qPCR (gDNA digester plus) kit (Yeasen Biotechnology Co., Ltd., Shenzhen, China). Specific primers were used for the RT-PCR, and α-tubulin-F/R was used as the internal control (Table 1). All PCR products were then analyzed through 1% agarose gel electrophoresis.

**TABLE 1:** Primers used in this study.

Primer name	Primer sequence (5′→3′)	Used for	Product size (bp)
CrtO-F CrtO-R	GTTCATCACCACCCACGACG CCGTAGTAGAACAGGCGGAA	PCR identification of *CrtO* gene	511
cDNA-CrtO-F cDNA-CrtO-R	CAGTTCCTGAAGATCGCCGTGT TAGCAGGTCAGGAAGCTCTGC	RT-PCR identification of *CrtO* gene	227
BKT2-F BKT2-R	CCTGAAGCACGCCTACAAG AGGTAGGTGCCGAAGTAGAA	PCR identification of *BKT2* gene	908
cDNA-BKT2-F cDNA-BKT2-R	GCCAGGTGAGCTACCGC CTTGTAGGCGTGCTTCAGGG	RT-PCR identification of *BKT2* gene	266
α-tubulin-F α-tubulin-R	CTCGCTTCGCTTTGACGGTG CGTGGTACGCCTTCTCGGC	Internal control for RT-PCR	132 (Balczun et al., 2005)

### 2.5 Chromatographic mass spectrometric analysis of carotenoids in transgenic algae

The collected algal cells were freeze-dried for 48 h and ground to a powder using a ball mill (30 Hz, 1 min). Then, a 50-mg powdered sample was weighed and 0.5 mL of a mixture of hexane/acetone/ethanol (1:1:1, v/v/v) containing 0.01% butylated hydroxytoluene (BHT) (g/mL) was added to extract the carotenoids. The concentrated extract was redissolved in 100 µL of a mixture of methanol/methyl tert-butyl ether (1:1, v/v) and filtered through a 0.22-µm organic membrane filter into a 1.5-mL amber centrifuge tube. The carotenoids were detected and quantified by liquid chromatography tandem mass spectrometry (LC-MS/MS; QTRAP 6500+, SCIEX) with a YMC C30 column (3 μm, 100 mm × 2.0 mm) (Krinsky et al., 2004). The basic chromatographic parameters were as follows: mobile phase A: methanol/acetonitrile (1:3, v/v) containing 0.01% BHT and 0.1% formic acid; mobile phase B: methyl tert-butyl ether containing 0.01% BHT; column temperature: 28°C; injection volume: 2 μL; flow rate: 0.8 mL/min. The gradient elution program was as follows: 0–3 min: 100% A and 0% B; 3–5 min: 30% A and 70% B; 5–10 min: 5% A and 95% B; 10–11 min: 100% A and 0% B. The mass spectrometry was performed as reported previously (Geyer et al., 2004) using an atmospheric pressure chemical ionization source (APCI) at 350°C and a curtain gas (CUR) pressure of 25 psi. In the QTRAP 6500+ spectrometer, each ion pair was scanned and detected according to the optimized declustering potential (DP) and collision energy (CE).

### 2.6 Qualitative and quantitative analyses of carotenoid products

A Metware database (MWDB) was constructed using standard compounds to qualitatively and quantitatively analyze the mass spectrometry data. Using the multiple reaction monitoring (MRM) method, the mass spectrometry data were extracted from multiple samples and converted to chromatographic peaks. The chromatographic peak areas of all detected samples were then substituted into the linear equation of the standard curve. The resulting values were then used in the carotenoid content calculation formula to ultimately determine the concentration of the substance in the actual sample.

$$Carotenoid\;content\;\left(µg/g\;DW\right)=c\;*\;V/1000/m$$

where c is the concentration (µg/mL) obtained by substituting the chromatographic peak area into the standard curve; V is the volume of solvent used during reconstitution (µL); m is the mass of the dried sample (g).

### 2.7 Statistical analysis

The metabolite content data were processed using unit variance (UV) scaling, and heatmaps were generated using the Complex Heatmap package in R software. The metabolomics data were analyzed from multiple perspectives by combining univariate statistical methods, including hypothesis testing and fold change (FC) analysis, with multivariate statistical methods. The differential metabolites were then screened based on the data characteristics using either the *p*-value or false discovery rate (FDR; for biological replicates ≥2) from the univariate analysis or FC value. The Kyoto encyclopedia of genes and genomes (KEGG) database was used for integrated analysis of the metabolite content. Pearson’s t-test was then used to compare two means.

## 3 Results

### 3.1 Effects of high light exposure on carotene metabolism in *C. reinhardtii*

**FIGURE 3:**
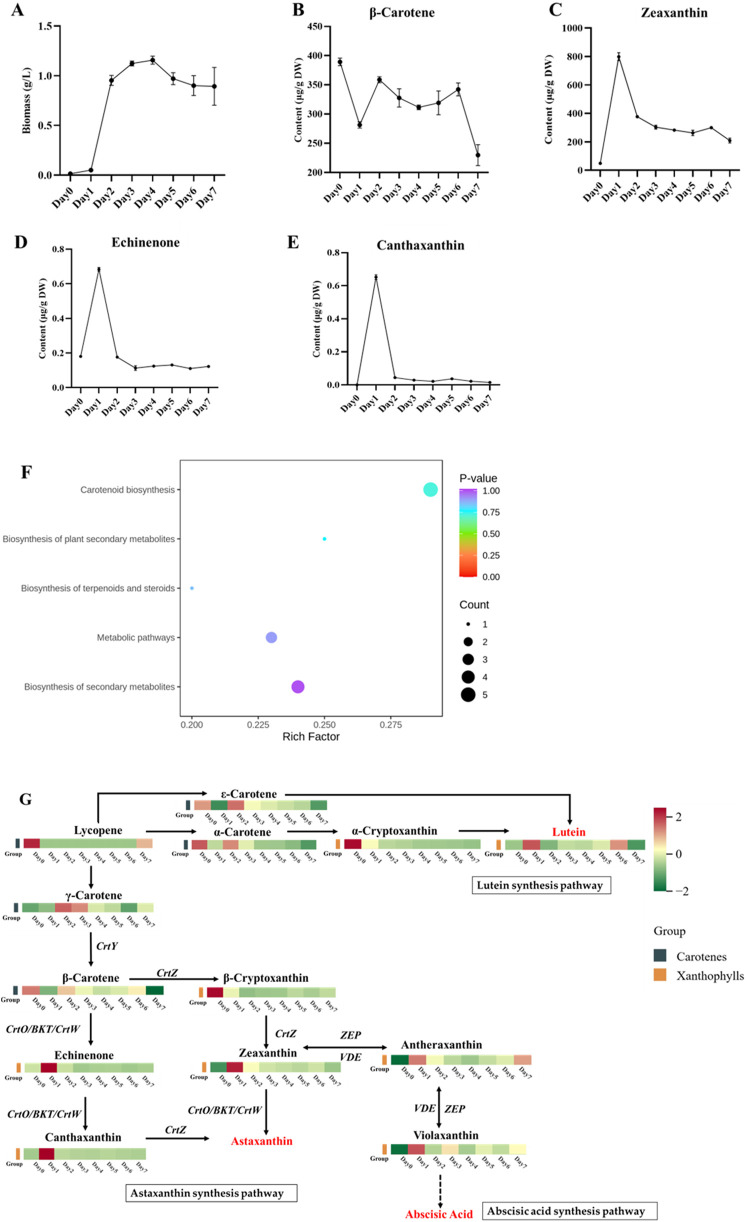
Changes in carotenoid metabolism in *C. reinhardtii* under stress caused by high-intensity light exposure. **(A)** Biomass of *C. reinhardtii* under high-intensity light stress for 7 days. **(B–E)** Dynamic changes in β-carotene, zeaxanthin, echinenone, and canthaxanthin biosyntheses in *C. reinhardtii* under high-intensity light stress. **(F)** KEGG enrichment analysis of the differentially expressed metabolites in *C. reinhardtii* in response to high-intensity light stress. The abscissa represents the Rich factor corresponding to each path, and the ordinate is the path name; the colors of the dots are the *p*-values, and red color indicates more significant enrichment. The sizes of the dots represent the numbers of enriched differential metabolites in the KEGG enrichment analysis chart of carotenoids. **(G)** Carotenoid biosynthesis pathway and heatmap representing the top-13 differentially expressed metabolites in *C. reinhardtii* in response to high-intensity light stress.

Carotenoids play an important role in the photosynthesis of microalgae. To study the changes in carotenoid components and content in *C. reinhardtii* under high light exposure, algal cells were treated with high-intensity light for 1 week. Starting from day 4, the proliferation of the algal cells entered a stable period, and the dry weight reached a maximum value of 1.15 g/L (Figure 3A). Under high-intensity light exposure, the β-carotene content decreased dramatically on day 1 and increased on day 2, followed by maintenance at a relative stable level till day 6. A dramatic drop was observed on day 7 to approximately 59% of that on day 0 (Figure 3B). Conversely, significantly higher content of zeaxanthin and echinenone at the maximal levels were observed on day 1, which were respectively 16.32 and 3.78 times of the content on day 0 (Figures 3C, D). Starting from day 2, the content of zeaxanthin and echinenone reduced significantly and were maintained relatively stable until day 7. The lowest level of zeaxanthin was found on day 7, which was still 4.27 times that observed on day 0. However, the lowest level of echinenone was observed on day3, which was only 67% of that noted on day 0. Moreover, canthaxanthin was almost undetectable on day 0 but increased to 0.65 μg/g on day 1 and was then maintained at 0.02–0.05 μg/g over days 2 to 7 (Figure 3E). Overall, high-intensity light stress negatively regulated the accumulation of β-carotene; positively induced the accumulation of zeaxanthin, echinenone, and canthaxanthin; and continuously promoted the accumulation of zeaxanthin and canthaxanthin in *C. reinhardtii*.

To further understand the metabolic changes under high-intensity light stress, a metabonomic analysis targeting carotenoids was performed using algal cells collected from days 0 to 7. A total of 32 metabolites were identified, of which 31 samples were found to be differential ones (Supplementary Table S1). The 31 differential metabolites were involved in five major metabolic pathways, namely, biosynthesis of carotenoids, biosynthesis of plant secondary metabolites, biosynthesis of terpenoids and steroids, metabolic pathways, and biosynthesis of secondary metabolites. In particular, the carotenoid biosynthesis pathway was found to be significantly enriched (Figure 3F).

The metabolic pathways recorded in the KEGG database for participation of the differential metabolites were also explored; this involved the analysis of 13 major differential metabolites, including five carotenes and eight xanthophylls. By mapping the differential metabolites in the carotenoid synthesis pathway, we found that the high-intensity light stress mainly affected the lutein, astaxanthin, and abscisic acid synthesis pathways (Figure 3G). The results showed that the content of carotenes (including α-carotene, γ-carotene, β-carotene, and ε-carotene) initially decreased on day 1, increased to the highest level on day 2, and declined gradually thereafter. Similarly, the content of the two xanthophyll metabolites, β-cryptoxanthin, and α-cryptoxanthin also decreased on day 1 but were maintained at levels lower than those on day 0 thereafter. However, the content of the remaining carotene (lycopene) and six xanthophylls accumulated and reached maximal levels on day 1 but decreased thereafter (Figure 3G). Briefly, the content of antheraxanthin and violaxanthin in the abscisic acid pathway decreased, while the content of echinenone and canthaxanthin in the astaxanthin synthesis pathway increased (Figure 3G). These results indicate that the stress caused by high-intensity light exposure promotes carotenoid metabolism in *C. reinhardtii* and stimulates the conversion of β-carotene to downstream substances, such as astaxanthin biosynthesis.

### 3.2 Heteroexpression of β-carotene ketolase in *C. reinhardtii*

To study the effects of β-carotene ketolases on canthaxanthin biosynthesis in *C. reinhardtii*, *CrtO* derived from *C. reinhardtii* and *BKT2* derived from *H. pluvialis* were employed and compared. Using the codon preferences of *C. reinhardtii*, the GC content of the *BKT2* gene was adjusted from 58% to 70%. The *RBCS2* intron 1 was inserted at the coding sequences of *CrtO* and *BKT2* to enhance gene expression (Figure 2). The *CrtO* and *BKT2* expression cassettes were inserted into the pH124 vector to obtain pH124-CrtO and pH124-BKT2, respectively, in which the target gene was driven by the *HSP70A/RBCS2* promoter (Figures 2A, B).

After transformation using the glass bead method (Li et al., 2021), a total of 122 transformants with a transformation efficiency of 4.01 × 10^−6^ and 52 transformants with an efficiency of 1.7 × 10^−6^ were obtained for pH124-CrtO and pH124-BKT2, respectively. The positive transformants with successful integration of the *CrtO* and *BKT2* genes were identified by genomic DNA PCR (Supplementary Figures S1A, B). The expressions of the β-carotene ketolase genes in the transformants were further confirmed by RT-PCR. The results suggested successful transcription of the β-carotene ketolase genes, as evidenced by the presence of specific bands of the *CrtO* and *BKT2* genes in the transformants as positive control (P) as well as absence of gene-specific bands in the negative control (wild type or WT) and template-free control (H_2_O) (Supplementary Figures S2A, B).

### 3.3 Effects of different β-carotene ketolases on carotenoid metabolism in *C. reinhardtii*

**FIGURE 4:**
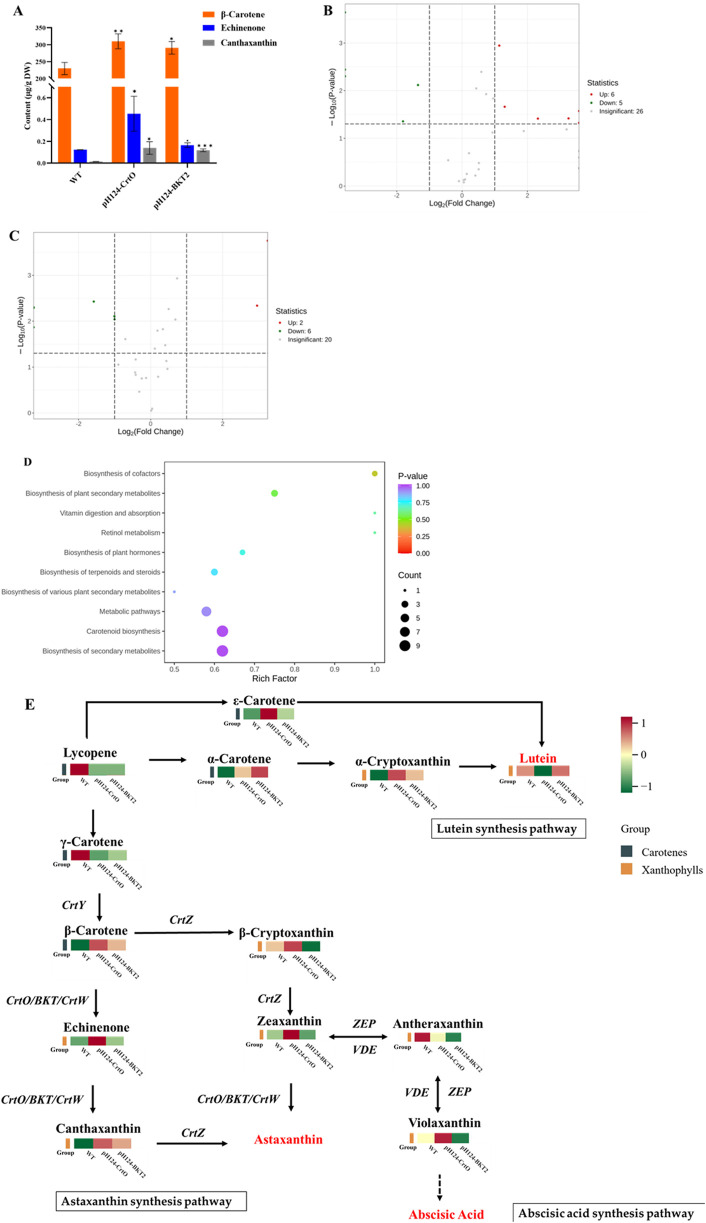
Carotenoid metabolisms in *C. reinhardtii* overexpressing β-carotene ketolases grown under high-intensity light stress. **(A)** Content of β-carotene, echinenone, and canthaxanthin in pH124-CrtO, pH124-BKT2, and wild type (WT). *, **, and *** indicate the significances between two means at the 0.01, 0.05, and 0.001 levels, respectively. **(B)** Differential metabolites in pH124-CrtO compared with WT. **(C)** Differential metabolites in pH124-BKT2 compared with WT. **(D)** KEGG enrichment analysis of the differentially expressed metabolites in *C. reinhardtii* in response to high-intensity light stress. The abscissa represents the Rich factor corresponding to each path, and the ordinate is the path name; the colors of the dots are the *p*-values, and red color indicates more significant enrichment. The sizes of the dots represent the numbers of enriched differential metabolites in the KEGG enrichment analysis chart of carotenoids. **(E)** Carotenoid biosynthesis pathway and heatmap representing the top-13 differentially expressed metabolites in pH124-CrtO, pH124-BKT2, and WT.

Qualitative and quantitative analyses of the carotenoid metabolites in the *C. reinhardtii* transformants of pH124-CrtO and pH124-BKT2 were performed using the LC-MS/MS technique with algal cells grown under high-intensity light stress for 7 d. The content of β-carotene, echinenone, and canthaxanthin were significantly higher in the pH124-CrtO and pH124-BKT2 transformants (Figure 4A). In detail, the content of β-carotene in pH124-CrtO and pH124-BKT2 were 310.28 μg/g and 290.97 μg/g, which were 1.35 and 1.27 times higher than that in the WT, respectively. The content of echinenone in pH124-CrtO and pH124-BKT2 were 0.45 μg/g and 0.17 μg/g, which were 3.75 and 1.42 times higher than that in the WT, respectively. The content of canthaxanthin in pH124-CrtO and pH124-BKT2 were 0.14 μg/g and 0.12 μg/g, which were 7 and 6 times higher than that in the WT, respectively.

The differences in metabolites among pH124-CrtO, pH124-BKT2, and WT were identified using thresholds of FC >2 and *p*-value <0.05 (Supplementary Tables S2, S3). The results show that a total of 37 metabolites were detected in pH124-CrtO vs. WT, including six increased and five decreased metabolites (Figure 4B). A total of 28 metabolites were detected in pH124-BKT2 vs. WT, including two increased and six decreased metabolites (Figure 4C). To further understand the metabolic changes under the presence of β-carotene ketolase, a metabonomic analysis targeting carotenoids was performed using algal cells collected from heterologous expressions of β-carotene ketolases. The results show that 37 differential metabolites were involved in ten major metabolic pathways, namely biosynthesis of cofactors, biosynthesis of plant secondary metabolites, vitamin digestion and absorption, retinol metabolism, biosynthesis of plant hormones, biosynthesis of terpenoids and steroids, biosynthesis of various plant secondary metabolites, metabolic pathways, biosynthesis of carotenoids, and biosynthesis of secondary metabolites (Figure 4D). Among these, the pathway involving biosynthesis of cofactors was significantly enriched. Subsequently, the 13 major differential metabolites (including five carotenes and eight xanthophylls) from among pH124-CrtO, pH124-BKT2, and WT were used in KEGG analysis to explore the associated metabolic pathways. Compared to WT, the major differential metabolites were mostly increased in the transformants, except for γ-carotene and antheraxanthin that were decreased in the transformants (Figure 4E). The content of antheraxanthin in the abscisic acid pathway decreased, while the content of α-carotene, β-carotene, α-cryptoxanthin, echinenone, and canthaxanthin in the astaxanthin synthesis pathway increased, indicating that the heteroexpressed β-carotene ketolases mainly affected the lutein, astaxanthin, and abscisic acid synthesis pathways (Figure 4E).

## 4 Discussion

It has been reported that high light exposure can increase the expression level of the lycopene β-cyclase gene, which is a key enzyme for β-carotene biosynthesis in microalgae (Ramos et al., 2008). Additionally, β-carotene ketolase (*BKT*), β-carotene hydroxylase (*CrtZ*), phytoene synthase (*PSY*), and phytoene desaturase (*PDS*) were upregulated in *H. pluvialis* under high-intensity light exposure (Gwak et al., 2014; He et al., 2018). Microalgae can successfully accumulate carotenoids under other stress conditions, such as nitrogen stress, phosphorus restriction, low temperatures, high salt content, etc. (Galasso et al., 2018). However, different microalgae exhibit different stress response mechanisms. For example, *Acutodesmus dimorphus* was reported to accumulate 75% neutral lipids among the total lipids under nitrogen-deficient conditions (Chokshi et al., 2017), and *C. reinhardtii* was shown to yield higher TAG levels under sulfur starvation (Sakarika and Kornaros, 2017). Under exposure to a light intensity of 170 μmol/m^2^/s for 2 d, the content of xanthophylls and β-carotene in *Tetraselmis* sp. CTP4 was reported to increase (Schüler et al., 2020). Similar findings were also observed in this study. We found that the carotenoid content in *C. reinhardtii* changed significantly under high-intensity light treatment, where the levels of the main metabolites were significantly different between the first and second days after treatment (Figure 3F). In addition, the content of the carotenoids varied in response to high-intensity light exposure at different stages of the microalgae. It has been reported that during the exponential period, high-intensity light exposure not only activates the gene expressions of the carotenoid synthesis pathways but also increases the proportion of carotenoids with photoprotective functions in the microalgae (Přibyl et al., 2016). During the stationary phase, strong light exposure induces transformation and accumulation of carotenoids to maintain a stable supply and accumulation of carotenoids within the cells, thereby helping the microalgae adapt better to high-intensity lit environments during the stable phase (Přibyl et al., 2016). Correspondingly, it was found that from days 1 to 2, when the microalgae were in the exponential phase, the overall content of β-carotene decreased while the content of zeaxanthin and echinenone increased. From days 3 to 7, when the microalgae were in the stationary phase, the levels of β-carotene, zeaxanthin, and echinenone tended to stabilize (Figure 3).

Several attempts were made in this study to improve the production of high-value canthaxanthin in *C. reinhardtii*. It has been reported that the presence of additional introns in the gene expression vector of *C. reinhardtii* can increase gene expression (Lumbreras et al., 1998). After insertion of *RbcS2* intron 1, the transformation efficiencies of the selection marker genes *Ble* and *Aph7*″ improved by 2–10 times over those with no intron insertion (Lumbreras et al., 1998). The *HSP70A/RBCS2* promoter and *PsaD* promoter can achieve high transgenic expression levels stably (Schroda, 2019). The *HSP70A/RBCS2* promoter has been reported to triple the expression of the *Ble* transgene (Schroda, 2019). Aside from the promoter, the terminator can also strongly impact transgene expression in *Chlamydomonas* (Schroda, 2019). When the *LUC* gene is driven by the same promoter with different terminators, the activity of the *LUC* reporter driven by the terminator of the *PsaD* gene increased by 40 times compared to the *β*_*2*_ *TUB* and *CCP1* genes (Kumar et al., 2013). Therefore, strong promoters, including *HSP70A/RBCS2*, were selected in this study to construct the β-carotene ketolase expression vector with additional introns and chloroplast guiding peptides (Figure 2). The results show that the content of β-carotene increased in the transformants generated by the *HSP70A/RBCS2* promoters coupled with introns (Figure 4E). Moreover, the increased content of carotenoids and lutein in these transformants indicate promotion of the carotenoid metabolites to downstream products (Supplementary Table S4).

Based on previous studies, β-carotene ketolases from different organisms can have different performances on astaxanthin production (Galasso et al., 2018). The *CrtW-*type β-carotene ketolase (mainly found in bacteria) symmetrically introduces ketogroups and modifies two β-rings at the same time, whereas the *CrtO*-type β-carotene ketolase (mainly found in cyanobacteria) only modifies one of the β-rings to produce echinenone (Fernández-González et al., 1997). Despite belonging to different classifications, the proteins encoded by the *BKT* gene from algae, *CrtW* gene from *Cyanobacteria* and bacteria, and *CrtO* gene from cyanobacteria (*Synechocystis* sp. *PCC 6803* and *Phormidesmis pristleyi*) appear to have similar functions in ketocarotenoid biosynthesis (Fernández-González et al., 1997; Rebelo et al., 2020). Related research has shown that *CrBKT* and *PrBKT* driving of *C. reinhardtii* CC125 can be used to explore the effects of overexpression and metabolic pathway changes of the colonies; the results show that overexpression of *PrBKT* in *C. reinhardtii* causes upregulation of more genes in the carotenoid pathway metabolism (Chen et al., 2023). Therefore, we selected β-carotene ketolases from different classifications, including *CrtO* from *C. reinhardtii* and *BKT2* from *H. pluvialis*, to find the best β-carotene ketolase for canthaxanthin production in *C. reinhardtii*. The metabolic analysis revealed that canthaxanthin accumulated by the *CrtO* and *BKT2* genes were 7 and 6 times higher than that in WT, respectively (Figure 4A). Further, *CrtO* accumulated more total carotenoid metabolites than *BKT2*, according to the carotenoid metabolomic analysis (Supplementary Table S4).

Genetic modifications for producing ketocarotenoids have been reported in yeast, bacteria, plants, and microalgae (Mortimer et al., 2016). The expression of *CrtW* in *E. coli* strains produced a high titer of canthaxanthin and 11.7 mg/g of total carotenoid, of which astaxanthin accounted for approximately 91% (Scaife et al., 2012). When *CrBKT* and *HpBHY* were coexpressed in tomato, the free astaxanthin in tomato leaves reached 3.12 mg/g, while the esterified astaxanthin in tomato fruit reached 16.1 mg/g (Huang et al., 2013). Overexpression of *CrBKT* and *PrBKT* driven by the strong *CaMV 35S* promoter in *C. reinhardtii* CC125 were shown to increase the total carotenoid content by 2.13 and 2.20 times, respectively. In particular, *CrBKT* and *PrBKT* increased the levels of β-carotene by 1.84 and 1.11 times, respectively (Chen et al., 2023). Similar findings were observed in the present study, where the content of β-carotene in the pH124-CrtO and pH124-BKT2 transformants increased by 1.35 and 1.27 times, respectively. Another study showed overexpression of *CrBKT* in the chloroplasts of *Nannochloropsis oceanica* and that combination with further metabolic engineering strategies such as enhanced carotenoid flux or inhibited competitive pathways can significantly increase canthaxanthin to about 4.7 mg/g dry weight (Liu et al., 2024). Overexpression of *CrBKT* driven by the strongly constitutive *HSP70/RBCS2* promoter in *C. reinhardtii* CC-4102 produced canthaxanthin under heterotrophic and dark conditions, where the yield of canthaxanthin reached 4.3 mg/L/d (Tran and Kaldenhoff, 2020). Additionally, the content of zeaxanthin and β-carotene were significantly decreased, while astaxanthin and canthaxanthin were successfully detected (Perozeni et al., 2020). In this study, the overexpression of β-carotene ketolases from different organisms in *C. reinhardtii* were shown to alter the carotenoid metabolism and increase the content of canthaxanthin (Figure 4). The transgenic *C. reinhardtii* strains expressing *CrtO* and *BKT2* had increased carotenoid content after high-intensity light exposure for 7 d. Unfortunately, astaxanthin was not detected. Although the content of canthaxanthin in the transformants was about 7 times that in the WT, the actual amount was 0.12–0.14 μg/g dry weight, which was still too low for astaxanthin accumulation. Astaxanthin can be produced in two ways using either canthaxanthin or zeaxanthin as the substrate. Since zeaxanthin is also a product of abscisic acid metabolism, there may be competition between the astaxanthin biosynthesis pathway and abscisic acid metabolism, resulting in astaxanthin production failure.

In summary, the major carotenoids accumulated in *C. reinhardtii* grown under high-intensity light stress were β-carotene followed by zeaxanthin. The introduction of β-carotene ketolase into *C. reinhardtii* dramatically enhanced canthaxanthin production. However, no astaxanthin was detected, possibly because of the competition between the abscisic acid metabolism and astaxanthin biosynthesis pathways, which is evidenced by the increases in violaxanthin. In the future, a mutant with defective zeaxanthin biosynthesis can possibly be used to solve the problem of substrate competition. Other genetic improvement strategies, such as optimization of the culture conditions, selection of suitable promoters, and introduction of high-efficiency enzymes, can also be attempted.

## 5 Conclusion

In this study, we evaluated the dynamic changes in carotenoid metabolism in *C. reinhardtii* under high-intensity light stress and modified canthaxanthin production by overexpressing β-carotene ketolase. The growth state of *C. reinhardtii* was not affected under high light exposure, and the dry weight reached its peak value on the fourth day. The metabolomics results showed that high light exposure promoted the synthesis of carotenoids in *C. reinhardtii* CC849, improved the intermediates of the astaxanthin synthesis pathway, and promoted the conversion of β-carotene to downstream substances. Metabolomics analyses of the carotenoid content were performed on the pH124-CrtO and pH124-BKT2 transformants, whose results showed that engineered strains of *C. reinhardtii* with increased canthaxanthin content were constructed successfully. Among these, the converters accumulate more carotene and canthaxanthin. The results showed that high light exposure and β-carotene ketolase genes increased the intermediate content of the astaxanthin pathway by reducing the intermediate content of the abscisic acid pathway, thereby promoting more carbon sources to flow to the astaxanthin pathway. Overall, the present study provides new insights into the synthesis of carotenoids through genetic engineering.

## Data Availability

The datasets presented in this study can be found in online repositories. The names of the repositories and accession numbers can be found in the article/Supplementary Material.
