# Supplementary material for: Effects of high light exposure and heterologous expression of β-carotene ketolase on the metabolism of carotenoids in Chlamydomonas reinhardtii
Source: Front Bioeng Biotechnol. 2025 Mar 10;13:1533661. doi: 10.3389/fbioe.2025.1533661 (PMC11938120; doi:10.3389/fbioe.2025.1533661)
Supplement: Supplementary file 2 [file DataSheet1.docx]

Supplementary Material

# Supplementary Figures


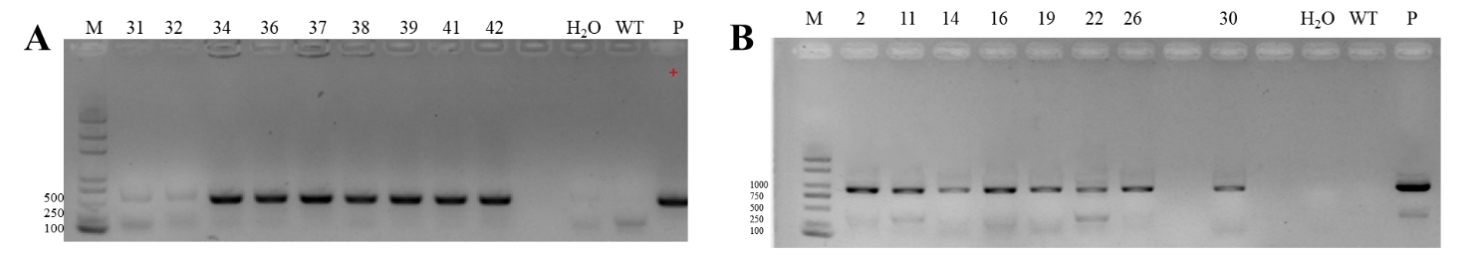


**Supplementary Figure 1.** Screening of transgenic *Chlamydomonas reinhardtii* by genomic DNA PCR. (A) PCR identification of *CrtO* gene. (B) PCR identification of *BKT2* gene. Lane M is DL5000 marker; Lanes 31~42 in (A) were transgenic individuals from pH124-CrtO; Lines 2~30 in (B) were transgenic individuals from pH124-BKT2; P was the positive control using plasmid DNA as the PCR template; and H_2_O was the no template control using sterilized ddH_2_O as the PCR template.


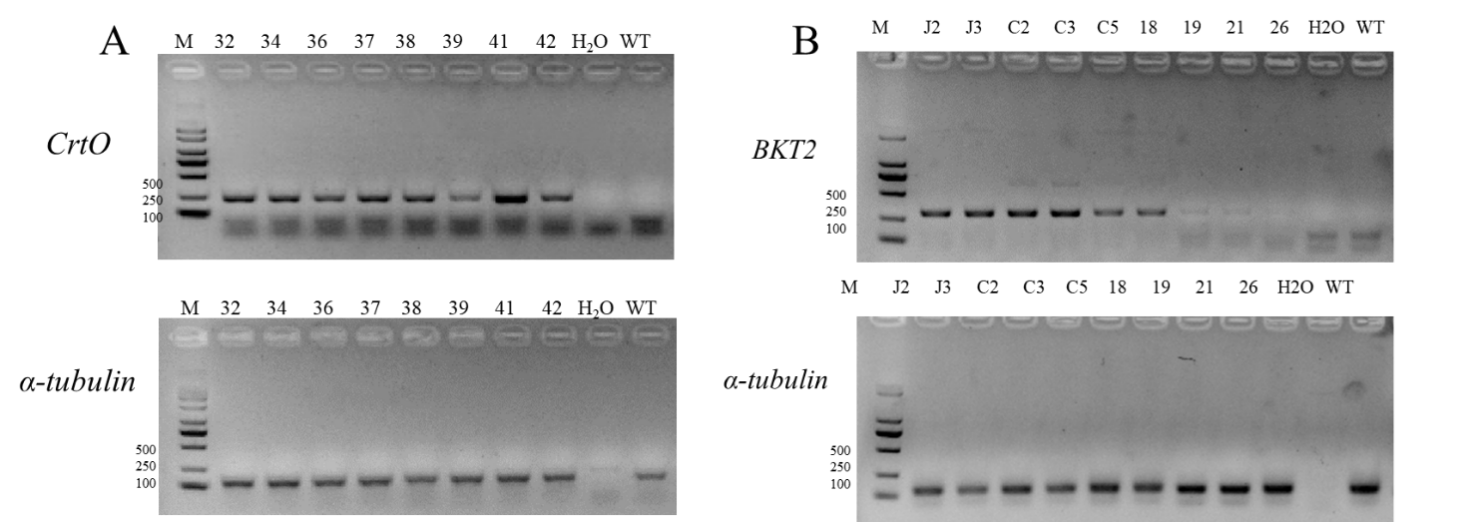


**Supplementary Figure 2.** Screening of transgenic *Chlamydomonas reinhardtii* by RT-PCR. (A) RT-PCR identification of *CrtO* gene. (B) RT-PCR identification of *BKT2* gene. Lane M is DL5000 marker; Lanes 31~42 in (A) were transgenic individuals from pH124-CrtO; Lines 2~30 in (B) were transgenic individuals from pH124-BKT2; P was the positive control using plasmid DNA as the PCR template; and H_2_O was the no template control using sterilized ddH_2_O as the PCR template. The expression of α-tubulin was served as the normalized control for RT-PCR.
